# Semiparametric approach to characterize unique gene expression trajectories across time

**DOI:** 10.1186/1471-2164-7-233

**Published:** 2006-09-13

**Authors:** Sandra L Rodriguez-Zas, Bruce R Southey, Charles W Whitfield, Gene E Robinson

**Affiliations:** 1 Department of Animal Sciences, University of Illinois at Urbana-Champaign, IL 61801, USA; 2 Department of Statistics, University of Illinois at Urbana-Champaign, IL 61801, USA; 3 Institute for Genomic Biology, University of Illinois at Urbana-Champaign, IL 61801, USA; 4 Department of Chemistry, University of Illinois at Urbana-Champaign, IL 61801, USA; 5 Department of Computer Science, University of Illinois at Urbana-Champaign, IL 61801, USA; 6 Department of Entomology, University of Illinois at Urbana-Champaign, IL 61801, USA

## Abstract

**Background:**

A semiparametric approach was used to identify groups of cDNAs and genes with distinct expression profiles across time and overcome the limitations of clustering to identify groups. The semiparametric approach allows the generalization of mixtures of distributions while making no specific parametric assumptions about the distribution of the hidden heterogeneity of the cDNAs. The semiparametric approach was applied to study gene expression in the brains of *Apis mellifera ligustica *honey bees raised in two colonies (*A. m. mellifera *and *ligustica*) with consistent patterns across five maturation ages.

**Results:**

The semiparametric approach provided unambiguous criteria to detect groups of genes, trajectories and probability of gene membership to groups. The semiparametric results were cross-validated in both colony data sets. Gene Ontology analysis enhanced by genome annotation helped to confirm the semiparametric results and revealed that most genes with similar or related neurobiological function were assigned to the same group or groups with similar trajectories. Ten groups of genes were identified and nine groups had highly similar trajectories in both data sets. Differences in the trajectory of the reminder group were consistent with reports of accelerated maturation in *ligustica *colonies compared to *mellifera *colonies.

**Conclusion:**

The combination of microarray technology, genomic information and semiparametric analysis provided insights into the genomic plasticity and gene networks linked to behavioral maturation in the honey bee.

## Background

The identification of collections of genes with unique or distinctive patterns of expression across time can enhance the understanding of gene pathways and time-dependent functional or biological processes like maturation. However, the number of collections (groups or clusters) of genes with distinctive pattern, expected gene expression trajectory within collection and probability of membership of each gene to each collection are unknown. The approach typically used to learn about all these unknowns involves two steps. First, the gene expression is described (typically using linear models) and point estimates (e.g. expected value) of the expression at each age are obtained. Second, these point estimates are used to assign genes to clusters using one of the many clustering approaches available (e.g. hierarchical clustering, k-means). Two major limitations of this approach are the limited information used to cluster the genes that may lead to bias in the final clustering and, the challenging identification of the optimal number of clusters. There are no completely satisfactory methods for determining the number of clusters [1]. The difficulties in identifying the cluster number suitable for each data set stem from the ambiguity and inconsistency of the indicators of cluster number and uncertain statistical properties of these indicators in a particular data set.

Mixture model approaches have been proposed to overcome some of the limitations of clustering. Finite mixture models have been applied to microarray data to profile the gene expression of independent discrete conditions (for example, tissue samples, tumor samples) or treatments. The clustering of gene expression patterns using gene expression from limited sample types is particularly challenging because the number of sample types is typically much smaller than the number of genes. Ghosh and Chinnaiyan [2] proposed a mixture model-based approach to classify genes based on the expression of independent samples corresponding to melanoma or prostate cancer diagnoses using a finite mixture of multivariate Normal distributions. McLachlan et al. [3] presented a model-based approach to cluster microarray gene expression data from independent tissue samples corresponding to colon and leukaemia cancer diagnoses. A mixture of t-distributions was used as a dimension reduction tool to reduce the number of genes to be used in clustering and to classify genes into classes. Alexandridis et al. [4] also applied finite mixture modeling to identify classes of genes based on gene expression from independent acute leukemia samples.

Most clustering approaches and the previously reviewed mixture approaches do not account for the dependencies of time-course gene expression data. Wu et al. [5] used a Markov chain model to account for the inherent dynamics of time-course gene expression patterns within a model-based clustering approach. However, this approach relies on converting the continuous gene expression levels into sequences of three discrete states (induced, repressed and constant) of expression. Nagin [6,7] described a semiparametric, group-based approach based on finite mixture modeling for analyzing behavioral development trajectories. This approach can be used to model directly continuous data, while providing estimates of group number, trajectories and group membership probability. The semiparametric group-based approach constitutes an example of an indirect application of finite mixture modeling because a finite number of groups is used to approximate a complex distribution. Direct applications of finite mixture modeling (for example [2,3] and [4]) assume a finite number of physically distinct groups [8]. The goal of this study was to evaluate the benefits of Nagin's [6,7] semiparametric, group-based approach to analyze gene expression data, identify distinct expression profiles, and provide probability of gene membership to each profile.

Honey bee is a well-established model to study the changes in gene expression associated with age-related changes in behavior and to identify collections of genes related to maturation. Worker honey bees work in the hive for the first 2 to 3 weeks of adult life in a variety of tasks including brood care (nursing) and then shift to foraging for nectar and pollen for the remainder of their 4 to 6 week life [9,10]. Microarray studies have revealed extensive changes in brain gene expression associated with honey bee behavioral maturation [11,12] These studies used statistical models with discrete age classes to describe the changes in gene expression and cluster analysis to group genes according to their profile [11]. Polynomial regression models of gene expression trajectories across age can provide a more parsimonious description for measurements that are close in time.

We used a semiparametric group-based approach to describe continuous time-dependent gene expression data and identify groups of cDNAs (and associated genes) with distinctive patterns of expression during honey bee behavioral maturation. The adequacy of the approach was assessed by integrating statistical tools and gene annotations based on the honey bee genome. Three kinds of analyses were performed. First, a semiparametric group-based approach was applied to the expression of cDNAs in honey bee brains across age. Second, the performance of the semiparametric approach was cross-validated by comparing the groups, trajectories and cDNA assignments to each group in two independent data sets. The results from the semiparametric approach were also compared to the corresponding results from a two-step clustering approach. Third, we used the annotation of the genes from the honey bee genome and Gene Ontology information to further validate the groupings from the semiparametric approach. The integration of the gene annotations supported by the genome and reliable grouping of genes using a semiparametric approach was used to gain further understanding of the genes associated with neurobiological functions and behavior in honey bees.

## Results

### General considerations

After processing and analyzing the intensities from the *ligustica *(*L*) and *mellifera *(*M*) data sets, the *P*-values corresponding to the linear, quadratic and cubic orthogonal trends across age were obtained for 6,848 and 7,027 cDNAs in each data set respectively. Of these cDNAs, 252 cDNAs were only present in the *M *data set and 73 cDNAs were only present in the *L *data set. These small differences in cDNAs present in both data sets were due to the filtering that resulted in different cDNAs being excluded in both data sets independently. A total of 529 cDNAs were present in both data sets and had significant (P < 0.00001 or Bonferroni adjusted P < 0.05) linear, quadratic or cubic orthogonal trends on age. Of these 529 cDNAs, 304 were assigned to genes identified in the honey bee genome. Due to redundancies in the assignment of multiple cDNAs to single genes, the 304 cDNAs corresponded to 268 different genes. Based on genome information [12], Gene Ontology (GO) information was available for 174 of the 268 identified genes.

### Number of groups

Table 1 summarizes the values of indicators of the optimal number of groups (BIC, AIC and MSE) from the semiparametric approach for the *L *and *M *data sets. Values for more extreme group numbers are not presented because they do not depart from the trend depicted in the range presented. These indicators show a substantial improvement of the model fit with increasing number of groups of cDNAs. The optimal number of groups identified by the minimum BIC and AIC values was ten in both data sets (Table 1). As expected the MSE criterion favors higher group numbers, however the reduction in MSE decreased with an increasing number of groups such that the change in MSE after 10 groups was minimal. The Pseudo-F criterion in the two-step clustering approach was ambiguous and showed several local maxima, one of them at ten clusters. The R^2 ^criteria did not provide further insight on the optimal number of clusters. Based on the semiparametric results, the trajectories from ten clusters are presented here.

### Description of main patterns

In the semiparametric approach, the cDNAs were assigned to the group with the highest probability of membership. The assignment of cDNAs to groups was consistent in the *L *and *M *data sets. Only 12 out of the 529 cDNAs were assigned to different groups between data sets and of these, 11 were assigned to a neighbor (closest) group with similar pattern (ascending or descending). Only one cDNA was assigned to two neighbor groups (2 and 3) that have different pattern of expression in both data sets.

**Figure 1:**
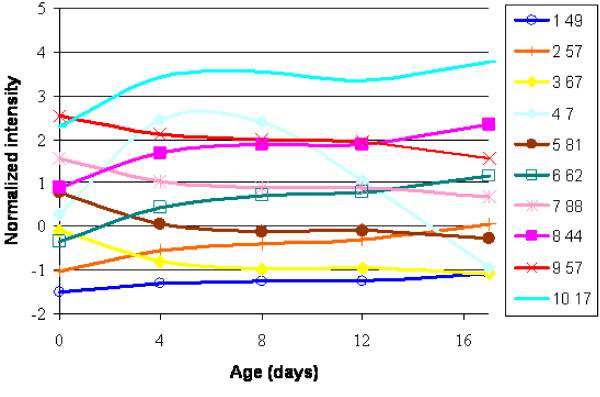
**Expected cDNA expression trajectories for each of the 10 groups identified in the ligustica (L) data set**. The legend indicates the group number and the estimated number of cDNAs per group.

**Figure 2:**
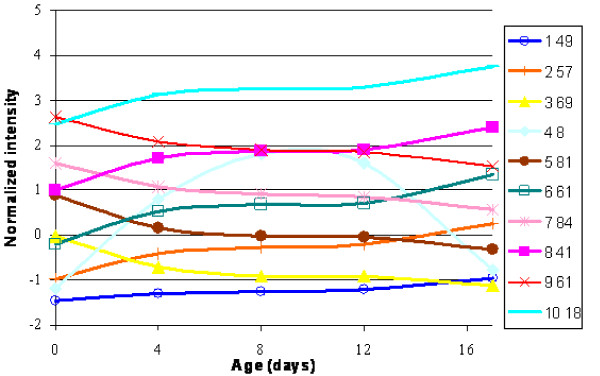
**Expected cDNA expression trajectories for each of the 10 groups identified in the mellifera (M) data set**. The legend indicates the group number and the estimated number of cDNAs per group.

**Figure 3:**
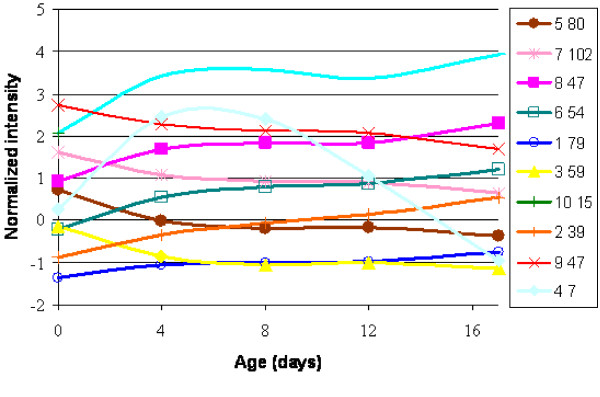
**Expected cDNA expression trajectories for each of the 10 clusters identified in the ligustica (L) data set using a clustering approach**. The legend indicates the group number and the estimated number of cDNAs per group.

**Figure 4:**
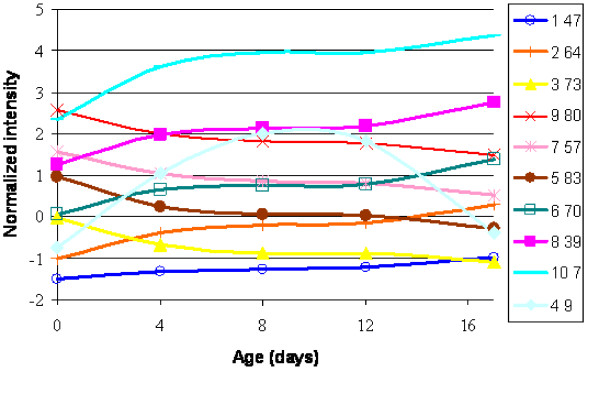
**Expected cDNA expression trajectories for each of the 10 clusters identified in the mellifera (M) data set using a clustering approach**. The legend indicates the group number and the estimated number of cDNAs per group.

The expected trajectories of the ten cDNA groups identified by the semiparametric approach in the *L *and *M *data sets are presented in Figures 1 and 2, respectively, and the ten clusters identified in the two-step clustering approach are presented in Figures 3 and 4, respectively. The pattern of Group 4 is different from all other groups and the remaining groups form two major trends that are essentially opposite to each other. The first major trend includes groups 3, 5, 7 and 9 that showed higher expression at day 0 than at all other days and a tendency for day 17 foragers to have the lowest expression levels. The second major trend includes groups 1, 2, 6, and 8 that showed the lowest expression at day 0 than at all other ages and a tendency for day 17 foragers to have the highest expression levels. Although some of the expected group trajectories seem similar, results from principal component analysis (including eigenvalues and principal component weights of each age) indicated intrinsic differences between the cDNAs within group, further supporting the unique nature of the ten groups.

The expected trajectory of group 4 was different from the other groups and varied between the two data sets. Group 4 exhibited a quadratic expected trend in age across data sets however peak expression was reached at younger ages in the *L *data set. The peak of the trajectory would interpolate approximately to day 9 in the *M *data set and to day 5 d in the *L *data set.

The similarity of the trajectories across data sets was further confirmed by the similarity in the regression coefficients in the semiparametric approach (Table 2). In most cases the expected value of each coefficient from one data set was within one standard error unit of the value of the coefficient in the other data set and in few cases (groups 6, 8, and 10) the difference was slightly higher than one standard unit. The only group that has substantial different estimates between data sets was group 4. This was consistent with the difference in trajectories that group 4 exhibited in Figures 1 and 2. The 95% confidence interval limits of the expected trajectories for each group and data set are provided in Additional files 3 and 4

The similarity of groups in the same major trend is also evident from the coefficients of the model terms in the semiparametric approach (Table 2). These groups tended to differ only in the magnitude of the coefficient of the intercept and a smaller difference in the magnitude of the coefficient of the linear term. The opposite relationship between the two major trends is clearly identified by the difference in sign of the coefficients corresponding to the linear, quadratic and cubic terms between the trends.

### Comparison of group-based and clustering results

The two-stage clustering approach did not offer clear evidence of the number of clusters supported by the data although 10 was among the likely numbers of clusters. The results from two-stage clustering using 10 clusters were used also to facilitate the comparison of results across approaches. The number of cDNAs and associated genes in each semiparametric group and two-step cluster is given in Table 3. Of the 529 cDNAs studied, 88.3% and 85.6% of the cDNAs were consistently assigned to the same collection by the semiparametric and two-step approaches in the *M *and *L *data sets, respectively. When the gene membership encompassed the most proximal (neighbor) groups with similar patterns, then the percentage of cDNAs consistently assigned to the same group was 97.3% and 98.3% for the *M *and *L *data sets respectively.

### Genome-based interpretation of the gene groups

The availability of the honey bee genome allowed the assignment of genes to groups with distinctive trajectories. Approximately 50 to 60% of the cDNAs within a group have been assigned to genes in the honey bee genome. The exception to this was semiparametric group 4 which included almost all cDNAs with gene assignments. The multiple assignment of cDNA to genes resulted in the presence of some genes in multiple groups. For example, in the L data set out of the 304 known genes, 88.8% (238) were assigned to single groups, 10 were assigned twice to the same group (due to cDNA redundancy on the microarray and similar patterns of expression), one was assigned three times to the same group (due to cDNA redundancy and similar pattern of expression), 15 were assigned to two different groups, three were assigned to two groups (one group receiving one cDNA and the other two cDNAs) and one was assigned to four different groups. The genes with multiple cDNAs assigned to multiple groups were distributed in proximal groups with the same of trajectory and similar level of expression. In addition, these cDNAs tended to have moderate and similar probability of membership to the corresponding groups.

The number of genome annotated genes with Gene Ontology (GO) information in each semiparametric group is given in Table 3. Based on the assembly 2 of the honey bee genome, only 50% to 60% of the genes had inferred GO information. The exception to this was the semiparametric group 4 that contain no gene with GO information.

Considering genes with roles in neurobiological processes, the assignment of genes to groups was consistent with their roles. The identification, group membership, and brief description of associated molecular function or biological process of the 54 genes associated with neurobiological processes for the *M *and *L *data set are given in Additional file 1. Out of 24 genes with a wide range of synaptic functions, 18 genes were assigned to groups with decreasing expression from day 0 to day 17. All three *MAPKKK *(Mitogen-activated protein Kinase Kinase Kinase signaling) cascade related genes were assigned to groups with ascending expression profile from day 0 to day 17. All except one voltage-gated channel gene were assigned to groups with decrease in expression from day 0 to day 17. Six of the nine genes with neurogenesis function were assigned to groups with increasing expression with age. Contrary to this, 12 out of the 14 genes associated with neurotransmitter functions were in groups with decreasing expression profile across the ages studied and four out of six genes associated with the development of the neural system had lower expression at the end of the period studied. Of the 14 genes with memory and/or learning or behavior related function, 10 genes were in groups exhibiting a descending profile from day 0 to day 17. Of the 5 axonal related function genes assigned to groups, approximately half were groups with ascending pattern across time points. Two out of the three genes associated with mushroom body development had descending expression from day 0 to day 17. Other genes with predicted function grouped by the semiparametric approach are axonal related and vision related genes.

Whitfield et al. [11] provided a list of 50 cDNAs present in the 9K microarray that can be used to predict nurse or forager behavior and 36 of these cDNAs were confirmed by Cash et al. [12]. In this study, out of the 529 cDNAs with significant linear, quadratic and cubic trends profiled, 25 were among the list of 50 predictive cDNAs and all have trajectories consistent with the pair-wise differences reported by Whitfield et al. [11] and Cash et al. [12]. The identification of the 25 consistent cDNAs, estimated normalized cDNA expression at each of the five ages measured and the overall forager-to-nurse ratio reported by Whitfield et al. [11] are provided in Additional file 2

## Discussion

The semiparametric group-based approach allowed the estimation of the number of cDNA (or gene) groups that are optimally identifiable using an unambiguous statistical criterion like BIC. This indicator confers a considerable advantage to the semiparametric approach in comparison to the approximate statistics available in the clustering approach that typically show multiple optima across the number of clusters. The consistency of the semiparametric grouping of cDNAs across data sets demonstrates the reproducibility of the semiparametric technique. The assignment of genes with related neural function based on the honey bee genome to the same group or groups with similar profile further validated the appropriateness of the semiparametric results. Although the semiparametric and two-step clustering approaches provided consistent number of groups and similar expected trajectories, consideration must be given to the fact that the identification of ten clusters was based on the number of groups suggested by the semiparametric approach because the clustering approach did not provide a single optimal number of clusters. In addition, the semiparametric approach provided the probability of membership of each cDNA to each group however this information was not available in clustering. This information was critical to identify cDNAs with high and low certainty of group assignment. The few cDNAs in the microarray with significant age by colony and colony effects may explain the differences between data sets in assignment of some cDNAs to different semiparametric groups and age at peak expression in group 4.

An advantage of the semiparametric approach over the two-step clustering approach implemented in this study is the objective assignment of cDNA to groups using the posterior probabilities of membership to each group. A cDNA is assigned to the group that has the maximum posterior membership probability for that cDNA. Overall the median assignment probabilities for each group are high (0.87) and this high certainty in assignment may be due to the high information content of the data. The strong signal contained in the data with respect to the cDNA trajectory may be one reason for the similarity between the semiparametric and two-step clustering approaches in this study. The advantage of the semiparametric approach may be more evident in data sets with more cDNAs with intermediate membership probabilities.

The assignment of cDNA to groups based on maximum probability does not ensure unquestionable membership to any group. Most of the cDNAs that were assigned to different groups between data sets did not have a clearly high membership probability to a single group because two or more dominant probabilities were estimated. This variability in membership probabilities reflects the semiparametric nature of the approach in that there is a continuous distribution of trajectories approximated by the discrete number of groups. The mode of the membership probabilities was lower for the trajectories with intermediate levels and was associated with higher frequency of inconsistent cDNA assignment across approaches and data sets. Examples of this are the cross-classification between group 7 and the two surrounding groups 5 and 9 and between group 6 and surrounding groups 8 and 2 (Figures 1 and 2). Higher membership or assignment probabilities in the extreme (high or low average expression levels) profiles were associated with more consistent assignment of cDNAs across approaches in these groups and examples of this are groups 1 and 10.

In the two-step clustering approach, only the point estimates of the first stage are used in the second stage. Thus, the values used in the second stage are assumed to be known without uncertainty [14] and any variability in the first stage is not accounted for in the second stage. In addition, measurements of clustering adequacy based on resampling are computational demanding [15] meanwhile approximate criteria (e.g. Pseudo-F) are ambiguous. By providing the probabilities of membership of each cDNA to each group, the semiparametric approach allows the consideration of the uncertainty inherent when classifying cDNAs to groups. The similarity of the results for the majority of the cDNAs in both approaches suggests that the uncertainty in the information being clustered has small impact on the grouping of cDNAs in this study. This is likely to be due to the requirement for all cDNAs to have intensity measurements in all age and at least 75% of the measurements within age. For the minority of the cDNA that were assigned to different collections in both approaches, the probability of membership of each cDNA to each group available in the semiparametric approach offered unique insights on the likely true grouping.

This study only considered the cDNAs with significant linear, quadratic or cubic trajectories across a 17-day maturation period because a goal was to further study the genes exhibiting trends across age. The semiparametric approach can be used to group any cDNA regardless of trend or level of expression. A pilot semiparametric grouping of all cDNAs indicated that the cDNAs with no clear trends in age tend to form few and large groups with flat trajectories across age at different levels. This is because the cDNAs are expressed at fairly constant levels across time and can have a significant intercepts. However, the cDNA groups with nonsignificant fluctuation across age could effectively hide the main trajectories or groups of interest. It is likely that a proportion of the cDNAs considered non-significant in this study are really false negatives due to the threshold *P*-value used however, the detection of accurate patterns for these cDNAs would require an experiment with greater statistical power. Likewise a few cDNAs (approximately five) are likely to be false positives and would also be incorrectly grouped. Consequently the expected influence of these errors would increase the uncertainty of the groupings. The influence of these errors was greatly minimized in this study by using the confirmatory *M *and *L *data sets. The majority of the cDNAs that were significantly differentially expressed were assigned to the same group in both data sets suggesting a minimal impact of the false positive and negative errors in this study. Although the true grouping is unknown, examination of the posterior probabilities of membership to each group from the semiparametric approach could also identify these errors. The cDNAs with high posterior probabilities to belong to a group are less likely to be incorrectly assigned than cDNAs with similar posterior probabilities across two or more groups.

The semiparametric group-based approach provided robust assignment of cDNAs to groups with distinct overall trajectories and alleviated some to the limitations of the two-stage clustering approach. This semiparametric approach can also model the effect of time dependent and independent explanatory variables on the trajectories of the groups. Two major limitations of the semiparametric group-based approach, commonly found in finite mixture approaches are that the cDNAs are assumed to be independent and the components are all assumed to follow the same type of distribution (in our study, multivariate Normal).

Other studies have characterized or predicted behavioral stages (nurse, forager) in honey bees using gene expression data from cDNA microarrays. Whitfield et al. [11] provided a list of 50 cDNAs that can be used to predict nurse or forager behavior and 36 of these cDNAs were confirmed by Cash et al. [12]. In this study, out of the 529 cDNAs with significant linear, quadratic and cubic trends profiled, 25 were among the list of 50 predictive cDNAs and all have trajectories consistent with the pair-wise differences reported by Whitfield et al. [11] and Cash et al. [12]. More cDNAs in the list of 50 predictive cDNAs were found when the *P*-value threshold was relaxed and in all cases the trends in age were consistent with previous studies. In addition, the cDNA representing the *forager *gene reported by Ben-Shahar et al. [16,17] had a significant linear trajectory (P < 0.000011) with higher expression levels in foragers than nurses. Six out of the 25 cDNAs were assigned to different groups in both data sets although these groups have similar trajectories. The primary reason for the different grouping appeared to be colony effects rather than statistical methodology. The ability to discriminate between nurse and forager behavior depended on the data set and the ages considered. The detection of significant linear, quadratic and cubic trends illustrates the advantages of repeated measurement studies.

The use of gene annotations from the honey bee genome and associated GO information is essential to the confirmation of the main trends uncovered by the semiparametric approach. In particular, we studied the grouping of genes known to be associated with neurobiological processes, excluding carbohydrate metabolism, based on their distinctive patterns within group. Many of the genes and profiles presented in Additional file 1 confirm results from previous studies. For example, Grozinger et al. [18] reported that queen pheromone caused a significant change in expression of the Kruppel homolog 1 (*Kr-h1*) gene in the bee brain. This gene has been linked to motor axon guidance and synaptogenesis in fruit fly larvae. The change in the expression of voltage-gated ion channels could be related to the changes in the expression of the *malvolio *gene, which encodes a manganese transporter [16], because manganese ions could pass through the cell membrane via voltage-gated calcium channels. Our approach to study trajectories of gene expression also uncovered consistent patterns in multiple genes pertaining to the *MAPKKK *(Mitogen-activated protein Kinase Kinase Kinase) cascade. These findings prompt further studies of the association between honey bee behavioral maturation and the *MAPKKK *gene pathways. These pathways have been less studied in the honey bee, although there is evidence in other insects that this pathway regulates synapse architecture [19].

## Conclusion

The identification of collections of genes with distinct expression profiles is an important piece of the puzzle that is the study of gene networks. We confirmed the ability of a semiparametric approach to provide unambiguous criteria to detect groups of genes, trajectories and probability of gene membership to the groups in two data sets. Genome annotations further confirmed that genes with known neurobiological function were assigned to the same group or groups with similar trajectories. The characterization of gene networks associated to behavioral maturation was enhanced by the integration of semiparametric analysis and bioinformatics tools.

## Methods

### Data sets

Whitfield et al. [20] measured the levels of cDNA expression during behavioral maturation ages in the brains of honey bees from two races (*Apis mellifera mellifera *and *Apis mellifera ligustica*) raised in one of two colonies (*mellifera *and *ligustica*) representing two environments. Full sisters were used to create the two host colonies for each race. Within each combination of race and colony, three nurse bees were sampled on days 0, 4, 8, 12 and 17 after adult emergence and three forager bees were sampled on day 17 after emergence.

The expression of genes from individual brains was assessed using the double-spotted *Apis mellifera *brain 9K version 3.0 cDNA microarray using the protocols described by Whitfield et al. [11], Grozinger et al. [18] and Cash et al. [12]. The majority of the cDNAs in the microarray (5001 out of 8887 reporter cDNAs) have been mapped to 3610 individual genes in the honey bee genome assembly version 2. Based on the annotation of the genome, there is an approximately 38% redundancy of cDNAs to mapped genes and approximately 55% (1970 genes) of these mapped gene have Gene Ontology information.

The gene expression from each combination of bee race and colony in the experiment of Whitfield et al. [20] is available as separate loop designs consisting of 20 cDNA microarrays. This loop design maximized the direct comparisons between consecutive time points because samples at consecutive ages and at the first (day 0 nurse) and last (day 17 forager) ages were hybridized to the same array. From the full experiment two independent data sets, *ligustica *(*L*) and *mellifera *(*M*), were identified and used for cross-validation purposes in this study. The *L *data set included the measurements from *ligustica *bees raised in a *ligustica *colony and the *M *data set included the measurements from *ligustica *nurse bees raised in a *mellifera *colony. Each data set consisting of 20 microarrays was analyzed separately and considered an independent data set suitable to validate the results from the reminder data set because no bee was part of both data sets and a pilot analysis indicated that only 0.75% of the cDNAs had a significant (P-value < 0.00001) age by colony interaction and only 0.79% of cDNAs had a significant colony effect. Thus, we expect that at least 98% of the cDNAs will exhibit the same pattern in both data set and should be consistently assigned to the same gene collections in both data sets.

### Data processing

The same filtering and analysis procedures were conducted separately for each data set. The background subtracted fluorescence intensities were set to 1 when background was higher and foreground intensity and log2-transformed. Spot (or feature) intensities were filtered when a) the spots pertain to controls or other sequences (e.g. virus, suspected to be contaminated or present in high levels in hypopharyngeal glands) also excluded in Cash et al. [12], b) the spots were deemed of bad quality (and assigned a -100 flag) by the image analysis software (GenePix Pro 5.0 [21]), c) the spots had low signal (foreground intensities lower the 3 standard deviations of the background intensity within dye and array), d) the Cook distance of a spot intensity from the other intensity values from the same brain and dye was > 0.99 thus suggesting an inconsistent or unusual spot, e) and the median of all the intensities of a cDNA within dye and array was less than 300 [11]. After filtering the intensities from the duplicated spots on the same microarray were combined into one value, the average of the two spots when available or the value of a single spot remaining after filtering. Finally the following two gene-wise filtering criteria were applied: i) the presence of at least 75% of the spots per cDNA (at least 9 spots per cDNA out of a possible maximum of 12 spots in the arrays with hybridizations of consecutive time points or 16 spots in the arrays including day 0 nurse and forager day 17 samples), and ii) the availability of measurements at all ages.

The log2 intensity values were normalized using a linear-logarithmic transformation [22] and a general model was applied to estimate the trends of gene expression in age using the two-step approach described by Wolfinger et al. [23]. First, a linear model including the fixed effect of dye and the random effect of microarray was fitted across all cDNAs to adjust all measurements across dye and microarray combinations. Second, a linear mixed effects model including the fixed effect of dye and age and the random effect of microarray was used for each previously adjusted cDNA intensities to identify the cDNAs with significant orthogonal linear, quadratic and cubic trend across day 0, 4, 8 and 12 nurse and day 17 forager honey bees. The contrasts excluded day 17 nurse bees due to the similarity in age between the 17 day nurse and forager bees. A set of cDNAs that had significant (Bonferroni adjusted P-value < 0.05) linear or higher age trends in both data sets were identified. The adjusted age estimates of gene expression at day 0, 4, 8 and 12 in nurse bees and at day 17 in forager bees were obtained for each cDNA with significant linear, quadratic or cubic trends within data set.

The estimates of gene expression at each age were analyzed in each data set (see Additional files 5 and 6) using a semiparametric group-based mixture approach [6,7] that assigns cDNAs to groups with distinct trajectories based on the probability of belonging to that group. A two-step clustering approach was also implemented for comparison purposes.

### Semiparametric approach

A group-based regression approach [6,7] was used to identify groups of cDNAs with distinctive expression trajectories and, model the uncertainty of the trajectories and of the membership of cDNAs to groups. An infinite number of groups are assumed and thus the mixing distribution is nonparametric. Nonparametric methods, unlike parametric methods, do not make (or make very few) assumptions about the distribution of the observations. This property is desirable in the identification of groups of gene expression trajectories because the distribution is unknown. The limitation of a full nonparametric approach is the typically low power of results. The semiparametric approach offers a compromise because it relies on mildly strong assumptions, thus reducing the risk of misspecification while maintaining the precision [24]. The derivation of the semiparametric approach assumes a continuous distribution of the groups or cDNA trajectories that is approximated by a discrete function. The finite-group approximation results in a semiparametric maximum-likelihood approach [25,26].

This semiparametric model allows for the generalization of mixtures of distributions while making no specific parametric assumptions about the distribution of the hidden heterogeneity over the cDNAs. The semiparametric maximum likelihood estimators are described in detail by Land et al. [25] and Nagin and Tremblay [27] in the context of psychometric data. Briefly, let *y*_*ij *_denote the gene expression level of cDNA *i *(*i *= 1, ..., *n*) at age *j *(*j *= 1, ..., *J*) and ***y***_*i *_= (*y*_*i*1_, ..., *y*_*iJ*_) denote the vector of gene expression across age.

The probability density function corresponding to cDNA *i *is:

f(yi)=∑k=1KPr(Ci=k)fk(yi|βk,σ2)
 MathType@MTEF@5@5@+=feaafiart1ev1aaatCvAUfKttLearuWrP9MDH5MBPbIqV92AaeXatLxBI9gBaebbnrfifHhDYfgasaacH8akY=wiFfYdH8Gipec8Eeeu0xXdbba9frFj0=OqFfea0dXdd9vqai=hGuQ8kuc9pgc9s8qqaq=dirpe0xb9q8qiLsFr0=vr0=vr0dc8meaabaqaciaacaGaaeqabaqabeGadaaakeaacqWGMbGzcqGGOaakieqacqWF5bqEdaWgaaWcbaGaemyAaKgabeaakiabcMcaPiabg2da9maaqahabaacbiGae4huaaLae4NCai3aaeWaceaacqWGdbWqdaWgaaWcbaGaemyAaKgabeaakiabg2da9iabdUgaRbGaayjkaiaawMcaaiabdAgaMnaaBaaaleaacqWGRbWAaeqaaaqaaiabdUgaRjabg2da9iabigdaXaqaaiabdUealbqdcqGHris5aOWaaeWaceaacqWF5bqEdaWgaaWcbaGaemyAaKgabeaakiabcYha8HGabiab9j7aInaaBaaaleaacqqGRbWAaeqaaOGaeiilaWccciGaeW3Wdm3aaWbaaSqabeaacqaIYaGmaaaakiaawIcacaGLPaaaaaa@53A2@

where *f*_*k *_(**y**_*i*_|**β**_k_, *σ*^2^) is the *k *component densities of the mixture, *C*_*i *_is an indicator variable that denotes the component that cDNA *i *belongs to, and *Pr*(*C*_*i *_= *k*) is the probability of cDNA *i *to belong to the *k *group. Thus *f*(**y**_*i*_) is a mixture distribution with K components. In this study, *f*_*k *_(**y**_*i*_|**β**_k_, *σ*^2^) follows a multivariate Normal distribution:

*f*_*k *_(**y**_*i*_|**β**_k_, *σ*^2^)~MVN(**X**_*i*_**β**_*k*_, **I**_*J*_*σ*^2^)

where ***X***_*i *_is the design matrix relating the observations of cDNA *i *to the parameter vector ***β***_k_. To describe the cDNA expression trajectories, linear, quadratic and cubic trends on age were considered and the design vector corresponding to cDNA *i *and age *j*, is xij=(1,ageij,ageij2,ageij3)
 MathType@MTEF@5@5@+=feaafiart1ev1aaatCvAUfKttLearuWrP9MDH5MBPbIqV92AaeXatLxBI9gBaebbnrfifHhDYfgasaacH8akY=wiFfYdH8Gipec8Eeeu0xXdbba9frFj0=OqFfea0dXdd9vqai=hGuQ8kuc9pgc9s8qqaq=dirpe0xb9q8qiLsFr0=vr0=vr0dc8meaabaqaciaacaGaaeqabaqabeGadaaakeaaieqacqWF4baEdaWgaaWcbaGaemyAaKMaemOAaOgabeaakiabg2da9maabmGabaGaeGymaeJaeiilaWIaemyyaeMaem4zaCMaemyzau2aaSbaaSqaaiabdMgaPjabdQgaQbqabaGccqGGSaalcqWGHbqycqWGNbWzcqWGLbqzdaqhaaWcbaGaemyAaKMaemOAaOgabaGaeGOmaidaaOGaeiilaWIaemyyaeMaem4zaCMaemyzau2aa0baaSqaaiabdMgaPjabdQgaQbqaaiabiodaZaaaaOGaayjkaiaawMcaaaaa@4DCB@. Although the availability of information on five ages allows the evaluation of up to quartic trends, the estimates of this trend would be based on limited information and would be unreliably estimated. The estimates of **β**_*k *_= (*β*_0*k*_, *β*_1*k*_, *β*_2*k*_, *β*_3*k*_) are group-specific and provide K cDNA expression trajectories across age.

The unobservable discrete variable *C*_*i *_indicates the group membership or group that the cDNA *i *pertains. This variable can take any of *K *distinct values, each corresponding to a distinct cDNA expression trajectory and *Pr*(*C*_*i *_= *k*) is the mixing proportion or weight. In addition

∑k=1KPr(Ci=k)=1,0≤∑k=1KPr(Ci=k)≤1
 MathType@MTEF@5@5@+=feaafiart1ev1aaatCvAUfKttLearuWrP9MDH5MBPbIqV92AaeXatLxBI9gBaebbnrfifHhDYfgasaacH8akY=wiFfYdH8Gipec8Eeeu0xXdbba9frFj0=OqFfea0dXdd9vqai=hGuQ8kuc9pgc9s8qqaq=dirpe0xb9q8qiLsFr0=vr0=vr0dc8meaabaqaciaacaGaaeqabaqabeGadaaakeaafaqabeGabaaabaWaaabCaeaaieGacqWFqbaucqWFYbGCcqGGOaakcqWGdbWqdaWgaaWcbaGaemyAaKgabeaakiabg2da9iabdUgaRjabcMcaPiabg2da9iabigdaXiabcYcaSaWcbaGaem4AaSMaeyypa0JaeGymaedabaGaem4saSeaniabggHiLdaakeaacqaIWaamcqGHKjYOdaaeWbqaaiab=bfaqjabckhaYjabcIcaOiabdoeadnaaBaaaleaacqWGPbqAaeqaaOGaeyypa0Jaem4AaSMaeiykaKIaeyizImQaeGymaedaleaacqWGRbWAcqGH9aqpcqaIXaqmaeaacqWGlbWsa0GaeyyeIuoaaaaaaa@54E5@

and *Pr*(*C*_*i *_= *k*) follows a polychotomous multinomial (*K *degree) logistic distribution:

Pr(Ci=k)=exp⁡(θκ)∑k=1Kexp⁡(θκ)
 MathType@MTEF@5@5@+=feaafiart1ev1aaatCvAUfKttLearuWrP9MDH5MBPbIqV92AaeXatLxBI9gBaebbnrfifHhDYfgasaacH8akY=wiFfYdH8Gipec8Eeeu0xXdbba9frFj0=OqFfea0dXdd9vqai=hGuQ8kuc9pgc9s8qqaq=dirpe0xb9q8qiLsFr0=vr0=vr0dc8meaabaqaciaacaGaaeqabaqabeGadaaakeaaieGacqWFqbaucqGGYbGCcqGGOaakcqWGdbWqdaWgaaWcbaGaemyAaKgabeaakiabg2da9iabdUgaRjabcMcaPiabg2da9maalaaabaGagiyzauMaeiiEaGNaeiiCaaNaeiikaGccciGae4hUde3aaSbaaSqaaiab+P7aRbqabaGccqGGPaqkaeaadaaeWbqaaiGbcwgaLjabcIha4jabcchaWjabcIcaOiab+H7aXnaaBaaaleaacqGF6oWAaeqaaOGaeiykaKcaleaacqWGRbWAcqGH9aqpcqaIXaqmaeaacqWGlbWsa0GaeyyeIuoaaaaaaa@50C7@

To address the estimability problem, one group level (e.g. 1) is considered the baseline level. Thus, *θ *= (*θ_2_*,..., *θ*_*k*_..., *θ*_*K*_) and the estimated parameters are the log odds of membership in level k versus the baseline group.

Based on the regression coefficient estimates, the probability of observing each expression pattern is computed conditional on its belonging to each group. The cDNA is then assigned to the group with the highest probability of having generated the group pattern.

The likelihood is:

L(β1,⋯,βK,σ2,θ1,⋯,θK|yi)=∏i=1n∏k=1K[Pr(Ci=k)Pr(yi|βk,σ2)]
 MathType@MTEF@5@5@+=feaafiart1ev1aaatCvAUfKttLearuWrP9MDH5MBPbIqV92AaeXatLxBI9gBaebbnrfifHhDYfgasaacH8akY=wiFfYdH8Gipec8Eeeu0xXdbba9frFj0=OqFfea0dXdd9vqai=hGuQ8kuc9pgc9s8qqaq=dirpe0xb9q8qiLsFr0=vr0=vr0dc8meaabaqaciaacaGaaeqabaqabeGadaaakeaaieWacqWFmbatdaqadiqaaGGabiab+j7aInaaBaaaleaacqaIXaqmaeqaaOGaeiilaWIaeS47IWKaeiilaWIae4NSdi2aaSbaaSqaaiabdUealbqabaGccqGGSaaliiGacqqFdpWCdaahaaWcbeqaaiabikdaYaaakiabcYcaSiab9H7aXnaaBaaaleaacqaIXaqmaeqaaOGaeiilaWIaeS47IWKaeiilaWIae0hUde3aaSbaaSqaaiabdUealbqabaGccqGG8baFieqacqaF5bqEdaWgaaWcbaGaemyAaKgabeaaaOGaayjkaiaawMcaaiabg2da9maarahabaWaaebCaeaadaWadiqaaGqaciab7bfaqjab7jhaYnaabmGabaGaem4qam0aaSbaaSqaaiabdMgaPbqabaGccqGH9aqpcqWGRbWAaiaawIcacaGLPaaacqWEqbaucqWEYbGCdaqadiqaaiab8Lha5naaBaaaleaacqWGPbqAaeqaaOGaeiiFaWNae4NSdi2aaSbaaSqaaiabdUgaRbqabaGccqGGSaalcqqFdpWCdaahaaWcbeqaaiabikdaYaaaaOGaayjkaiaawMcaaaGaay5waiaaw2faaaWcbaGaem4AaSMaeyypa0JaeGymaedabaGaem4saSeaniabg+GivdaaleaacqWGPbqAcqGH9aqpcqaIXaqmaeaacqWGUbGBa0Gaey4dIunaaaa@74C3@

The Bayesian information criterion (BIC) was used to identify the optimal number of groups supported by the data [27,28].

The expression for BIC is:

BIC = -2 [log_e_(likelihood)] + plog(N)

where *log*_*e *_denotes the natural logarithmic transformation, *p *is the number of parameters in the model and *N *is the number of observations.

The BIC can consistently identify the optimal number of components in the mixture model [14] even when the models are not nested [29]. The BIC approach favors parsimonious models and Kass and Raftery [31] indicated that BIC can be used to approximate the Bayes factor for comparisons of models. The model with the smallest BIC value is preferred over the alternative specifications. The BIC offers a good compromise between model adequacy and simplicity when compared to the Akaike information criterion (AIC) and the mean square error (MSE) that tend to favor more and less parsimonious models than BIC respectively [30]. The parameters were estimated by direct maximization of the full likelihood using a SAS macro [32]. All models with 2 to 15 groups following a polynomial trajectory of up to order three were evaluated. The maximum number of groups considered was 15 because higher group numbers resulted in groups with less than 1% of the cDNAs studied within group and the trajectory would not be reliably estimated.

### Two-step clustering approach

In the two-step clustering approach, a cubic polynomial was fitted to the cDNA estimates across age and the predicted intensities at each age were clustered. Complementary clustering techniques were used to collect the cDNAs into clusters. Within hierarchical clustering, a maximum-likelihood hierarchical clustering (or EML) method was implemented. In EML, the distance between clusters A and B is:

DAB=Nvlog⁡e(1+[(∑i‖xi−x¯A‖2+∑i‖xi−x¯B‖2−∑i‖xi−x¯C‖2)/(∑J=1G‖xi−x¯J‖2)])−2(NCln⁡(NC)−NAln⁡(NA)−NBln⁡(NB))
 MathType@MTEF@5@5@+=feaafiart1ev1aaatCvAUfKttLearuWrP9MDH5MBPbIqV92AaeXatLxBI9gBaebbnrfifHhDYfgasaacH8akY=wiFfYdH8Gipec8Eeeu0xXdbba9frFj0=OqFfea0dXdd9vqai=hGuQ8kuc9pgc9s8qqaq=dirpe0xb9q8qiLsFr0=vr0=vr0dc8meaabaqaciaacaGaaeqabaqabeGadaaakeaafaqadeGabaaabaGaeeiraq0aaSbaaSqaaiabbgeabjabbkeacbqabaGccqGH9aqpcqqGobGtcqqG2bGDcyGGSbaBcqGGVbWBcqGGNbWzdaWgaaWcbaGaeeyzaugabeaakiabcIcaOiabigdaXiabgUcaRiabcUfaBjabcIcaOmaaqafabaWaauWaceaacqqG4baEdaWgaaWcbaGaeeyAaKgabeaakiabgkHiTiqbbIha4zaaraWaaSbaaSqaaiabbgeabbqabaaakiaawMa7caGLkWoadaahaaWcbeqaaiabikdaYaaaaeaacqqGPbqAaeqaniabggHiLdGccqGHRaWkdaaeqbqaamaafmGabaGaeeiEaG3aaSbaaSqaaiabbMgaPbqabaGccqGHsislcuqG4baEgaqeamaaBaaaleaacqqGcbGqaeqaaaGccaGLjWUaayPcSdWaaWbaaSqabeaacqaIYaGmaaaabaGaeeyAaKgabeqdcqGHris5aOGaeyOeI0YaaabuaeaadaqbdiqaaiabbIha4naaBaaaleaacqqGPbqAaeqaaOGaeyOeI0IafeiEaGNbaebadaWgaaWcbaGaee4qameabeaaaOGaayzcSlaawQa7amaaCaaaleqabaGaeGOmaidaaaqaaiabbMgaPbqab0GaeyyeIuoakiabcMcaPiabc+caViabcIcaOmaaqahabaWaauWaceaacqqG4baEdaWgaaWcbaGaeeyAaKgabeaakiabgkHiTiqbbIha4zaaraWaaSbaaSqaaiabbQeakbqabaaakiaawMa7caGLkWoadaahaaWcbeqaaiabikdaYaaaaeaacqqGkbGscqGH9aqpcqaIXaqmaeaacqqGhbWra0GaeyyeIuoakiabcMcaPiabc2faDjabcMcaPaqaaiabgkHiTiabikdaYiabcIcaOiabb6eaonaaBaaaleaacqqGdbWqaeqaaOGagiiBaWMaeiOBa4MaeiikaGIaeeOta40aaSbaaSqaaiabboeadbqabaGccqGGPaqkcqGHsislcqqGobGtdaWgaaWcbaGaeeyqaeeabeaakiGbcYgaSjabc6gaUjabcIcaOiabb6eaonaaBaaaleaacqqGbbqqaeqaaOGaeiykaKIaeyOeI0IaeeOta40aaSbaaSqaaiabbkeacbqabaGccyGGSbaBcqGGUbGBcqGGOaakcqqGobGtdaWgaaWcbaGaeeOqaieabeaakiabcMcaPiabcMcaPaaaaaa@A3B1@

where

D_AB _= distance between clusters A and B,

N = number of observations (cDNAs),

v = number of variables (5 ages),

C = cluster resulting from grouping clusters A and B,

log_e _= natural logarithmic transformation,

N_J _= number of observations in cluster J (for J = 1, ..., A, ..., B, ..., C)

x¯J
 MathType@MTEF@5@5@+=feaafiart1ev1aaatCvAUfKttLearuWrP9MDH5MBPbIqV92AaeXatLxBI9gBaebbnrfifHhDYfgasaacH8akY=wiFfYdH8Gipec8Eeeu0xXdbba9frFj0=OqFfea0dXdd9vqai=hGuQ8kuc9pgc9s8qqaq=dirpe0xb9q8qiLsFr0=vr0=vr0dc8meaabaqaciaacaGaaeqabaqabeGadaaakeaacuqG4baEgaqeamaaBaaaleaacqqGkbGsaeqaaaaa@2F82@ = sample mean vector pertaining to cluster J

The EML clustering method clusters observations or clusters that maximizes the likelihood at each level of the dendrogram and assumes multivariate Normal mixture distribution, equal spherical covariance matrices and unequal sampling probabilities [33]. The EML approach does not have the bias towards clusters of equal number of cDNAs that the Ward's method has and may not be appropriate for this data set. This method was implemented using SAS [33].

The k-means clustering approach was also implemented and results were compared to hierarchical clustering to minimize the potential impact of the clustering method on the resulting groups. This approach requires the specification of the number of clusters and uses the minimum least squares criterion. Euclidean distances were used, and three sets of seeds were tested to minimize the impact of the starting values on the resulting clustering implemented using SAS [33]. The clustering approaches are suitable to group the cDNA expression trajectories because there was no evident outlier intensity estimates across age. Evidence of this was the lack of singleton clusters with one cDNA in the k-means clustering. The optimal number of clusters supported by the data was identified based on consensus on three criteria computed, the R^2 ^(proportion of the total variance accounted by the clusters), R^2 ^ratio [R^2^/(1-R^2^)], and the pseudo-F statistic:

***Pseudo F *= ⌊*R***^2^**/(*G *- 1)⌋/⌊(1 - *R***^2^**)/(*N *- *G*)⌋**

where

G = number of clusters and,

N = number of cDNAs.

The criteria used to identify the optimal number of clusters were local pseudo-F maxima and slight reductions of the R^2 ^indicators across cluster numbers. There was no single optimal number of clusters (multiple local pseudo-F maxima were detected) and the number closest to the number of groups identified with the semiparametric approach was used for comparison purposes. The outputs from both clustering approaches were highly consistent and therefore only the results of the hierarchical EML method applied to the *M *and *L *data sets are presented.

### Model performance

The performance of this approach was evaluated in three-ways. The results from both data sets were compared first to each other, second to the results from a less obvious two-step clustering analysis and third further validated using the gene annotations supported by the assembly version 2 of the honey bee genome [13] and associated gene ontology. The cDNA collections are termed "groups" and "clusters" to distinguish the results from the semiparametric and two-step clustering approaches, respectively.

## Authors' contributions

SRZ conceived the idea of using semiparametric approach to study gene expression patterns in the honey bee brains, implemented the approach and was the leader in drafting the manuscript. BRS participated in the data preparation and analysis, contributed to the interpretation of the results and to draft the manuscript. CWW designed the microarray experiment, collected the samples and obtained the microarray data. GER conceived the experiment, participated in the experimental design and contributed to draft the manuscript. All authors read and approved the final manuscript.

## Supplementary Material

Additional File 1 Apis mellifera (honey bee) gene identification (GB prefix), Drosophila (fruit fly) homologous (CG prefix), semiparametric group membership and Gene Ontology (GO) information of 54 genes with neurobiological function in the mellifera (M) data set and in the ligustica (L) data set if different from M. This table provides the functional classification, honeybee gene identification, corresponding fruit fly gene identification, Gene Ontology description and, semiparametric group assignment in the mellifera (M) and ligustica (L) data sets of 54 genes with neurobiological function studied. Click here for file

Additional File 2 List of 25 cDNAs with significant (P < 0.00001) linear or quadratic or cubic profiles from day 0 (nurse) to day 17 (forager), estimate of expression at each day in the ligustica (L) and mellifera (M) data sets, gene group, and the relative expression between forager and nurse (FvsN) bees reported by Whitfield et al. (2003). This table provides a list with the identification of 25 cDNAs with significant linear, quadratic or cubic trends across days, the estimated gene expression at each day, semiparametric group, and the relative expression between forager and nurse bees reported in Whitfield et al. (2003) for the L and M data sets. Click here for file

Additional File 3 Confidence interval limits (95%) of the expected cDNA expression trajectories for each of the 10 groups identified in the ligustica (L) data set. The legend indicates the group number and lower (lc) or upper (uc) confidence interval limits per group. The figure presents the 95% confidence interval limits of the expected cDNA expression trajectories for each of the 10 groups identified in the L data set. Click here for file

Additional File 4 Confidence interval limits (95%) of the expected cDNA expression trajectories for each of the 10 groups identified in the mellifera (M) data set. The legend indicates the group number and lower (lc) or upper (uc) confidence interval limits per group. The figure presents the 95% confidence interval limits of the expected cDNA expression trajectories for each of the 10 groups identified in the M data set. Click here for file

Additional File 5 L data set. L data set estimates per cDNA and age. Click here for file

Additional File 6 M data set. M data set estimates per cDNA and age. Click here for file

## Figures and Tables

**Table 1:** Summary of Bayesian information criterion (BIC), Akaike information criterion (AIC) and means square error (MSE) by number of groups for the mellifera and ligustica data sets.

Data set						
	*mellifera*			*ligustica*		

Groups	BIC	AIC	MSE	BIC	AIC	MSE

5	-3103.31	-3049.92	0.586	-3065.63	-3012.24	0.586
6	-2964.67	-2900.60	0.526	-2961.00	-2896.94	0.526
7	-2894.63	-2819.89	0.493	-2902.09	-2827.35	0.493
8	-2843.36	-2757.94	0.466	-2766.41	-2851.83	0.466
9	-2727.89	-2633.44	0.432	-2737.53	-2641.43	0.443
10	-2637.40	-2530.63	0.409	-2665.38	-2558.61	0.416
11	-2668.76	-2551.31	0.403	-2679.47	-2562.02	0.406

**Table 2:** Estimates, standard error (SE) and P-value of the intercept, linear, quadratic and cubic terms describing the 10 groups using a semiparametric approach in the ligustica and mellifera data sets.

		Intercept			Linear			Quadratic			Cubic		
Group	Dataset	Estimate	SE	P-value	Estimate	SE	P-value	Estimate	SE	P-value	Estimate	SE	P-value

1	*ligustica*	-1.4922	0.0774	<0.0001	0.0813	0.0403	0.044	-0.0092	0.0058	0.1144	0.0003	0.0002	0.122
	*mellifera*	-1.4635	0.0704	<0.0001	0.0685	0.0372	0.0654	-0.0078	0.0054	0.1541	0.0003	0.0002	0.1223
2	*ligustica*	-1.0266	0.0624	<0.0001	0.1773	0.0351	<0.0001	-0.017	0.0052	0.0011	0.0006	0.0002	0.0025
	*mellifera*	-0.9844	0.0612	<0.0001	0.2214	0.0335	<0.0001	-0.0232	0.005	<0.0001	0.0009	0.0002	<0.0001
3	*ligustica*	-0.0964	0.0685	0.159	-0.2606	0.0339	<0.0001	0.025	0.0049	<0.0001	-0.0008	0.0002	0
	*mellifera*	-0.0233	0.0599	0.6969	-0.2479	0.0307	<0.0001	0.0231	0.0046	<0.0001	-0.0007	0.0002	<0.0001
4	*ligustica*	0.2749	0.1579	0.0818	0.9044	0.0956	<0.0001	-0.1	0.0144	<0.0001	0.0025	0.0006	<0.0001
	*mellifera*	-1.1858	0.1465	<0.0001	0.5999	0.0886	<0.0001	-0.023	0.0134	0.0864	-0.0006	0.0005	0.2179
5	*ligustica*	0.7813	0.0536	<0.0001	-0.2726	0.0289	<0.0001	0.027	0.0043	<0.0001	-0.0009	0.0002	<0.0001
	*mellifera*	0.9021	0.0553	<0.0001	-0.2764	0.0284	<0.0001	0.0272	0.0042	<0.0001	-0.0009	0.0002	<0.0001
6	*ligustica*	-0.3338	0.0702	<0.0001	0.2797	0.0328	<0.0001	-0.0255	0.0049	<0.0001	0.0008	0.0002	<0.0001
	*mellifera*	-0.1963	0.0593	0.0009	0.2989	0.033	<0.0001	-0.0337	0.0049	<0.0001	0.0013	0.0002	<0.0001
7	*ligustica*	1.5573	0.0526	<0.0001	-0.2004	0.0281	<0.0001	0.02	0.0042	<0.0001	-0.0007	0.0002	<0.0001
	*mellifera*	1.612	0.0588	<0.0001	-0.1945	0.0281	<0.0001	0.0183	0.0042	<0.0001	-0.0006	0.0002	0.0001
8	*ligustica*	0.8752	0.0685	<0.0001	0.3204	0.0391	<0.0001	-0.0336	0.0058	<0.0001	0.0012	0.0002	<0.0001
	*mellifera*	1.0052	0.0671	<0.0001	0.286	0.0394	<0.0001	-0.0309	0.0059	<0.0001	0.0011	0.0002	<0.0001
9	*ligustica*	2.5404	0.0607	<0.0001	-0.1607	0.0341	<0.0001	0.0164	0.0051	0.0013	-0.0006	0.0002	0.002
	*mellifera*	2.636	0.0569	<0.0001	-0.2106	0.0328	<0.0001	0.0204	0.0049	<0.0001	-0.0007	0.0002	0.0003
10	*ligustica*	2.2781	0.1012	<0.0001	0.4776	0.0615	<0.0001	-0.055	0.0092	<0.0001	0.0019	0.0004	<0.0001
	*mellifera*	2.4787	0.1008	<0.0001	0.2641	0.0606	<0.0001	-0.029	0.009	0.0013	0.0011	0.0004	0.0026

**Table 3:** Number cDNAs, corresponding genes based on the honey bee genome and genes with Gene Ontology (GO) information for each expression trajectory group assigned by the semiparametric and regression-clustering approaches in the mellifera and ligustica data sets.

	*mellifera*					*ligustica*				
	Semiparametric			Clustering		Semiparametric			Clustering	

Group	cDNA	Genes	GO	cDNA	Genes	cDNA	Genes	GO	cDNA	Genes

1	49	26	11	80	25	49	28	12	47	42
2	57	33	22	102	40	57	31	22	39	24
3	69	39	20	47	40	67	36	21	73	31
4	8	7	0	54	8	7	7	0	80	7
5	81	50	27	79	52	81	49	27	57	50
6	61	31	12	59	34	62	32	12	83	28
7	84	48	36	15	33	88	52	33	70	60
8	41	24	16	39	22	44	26	14	64	26
9	61	38	24	47	48	57	36	26	7	30
10	18	8	5	7	2	17	7	6	9	6

**Table 4:** Distribution of all 304 known genes and 173 genes with Gene Ontology (GO) information across semiparametric groups and regression clusters for the mellifera and ligustica data sets.

	*mellifera*			*ligustica*		
	Semiparametric		Clustering	Semiparametric		Clustering

Group	Gene	GO	Gene	Gene	GO	Gene

1	26	12	25	28	11	42
2	33	22	40	31	22	24
3	39	21	40	36	20	31
4	7	0	8	7	0	7
5	50	27	52	49	27	50
6	31	12	34	32	12	28
7	48	33	33	52	36	60
8	24	14	22	26	16	26
9	38	26	48	36	24	30
10	8	6	2	7	5	6
