# Supplementary material for: Semiparametric approach to characterize unique gene expression trajectories across time
Source: BMC Genomics. 2006 Sep 13;7:233. doi: 10.1186/1471-2164-7-233 (PMC1592090; doi:10.1186/1471-2164-7-233)
Supplement: Additional File 1 — Apis mellifera (honey bee) gene identification (GB prefix), Drosophila (fruit fly) homologous (CG prefix), semiparametric group membership and Gene Ontology (GO) information of 54 genes with neurobiological function in the mellifera (M) data set and in the ligustica (L) data set if different from M. This table provides the functional classification, honeybee gene identification, corresponding fruit fly gene identification, Gene Ontology description and, semiparametric group assignment in the mellifera (M) and ligustica (L) data sets of 54 genes with neurobiological function studied. [file 1471-2164-7-233-S1.doc]

| Functional classification | Bee Gene | Fly Gene | *M*  Group | Brief GO description | *L*  Group |
| --- | --- | --- | --- | --- | --- |
| Behavioral | GB11170-PA | CG6703 | 7 | adult walking behavior |  |
|  | GB14520-PA | CG3665 | 5 | Learning/memory, behavioral response to ethanol, olfactory learning |  |
|  | GB14772-PA | CG7524 | 3 | Learning/memory, olfactory learning |  |
|  | GB15257-PA | CG4917 | 9 | Sound perception |  |
|  | GB15582-PA | CG31196 | 9 | response to external stimulus/radiation, nonassociative learning |  |
|  | GB16014-PA | CG4700 | 9 | adult and flight behavior |  |
|  | GB16408-PA | CG8772 | 3 | memory, locomotion behavior |  |
|  | GB17817-PA | CG18069 | 5 | Learning/memory, courtship behavior |  |
|  | GB18814-PA | CG12348 | 5 | Taste perception, behavioral response to ether, courtship behavior, flight behavior |  |
|  | GB19979-PA | CG14994 | 7 | larval locomotion behavior |  |
|  | GB15327-PA | CG6917 | 2 | Courtship/mating behavior |  |
|  | GB11775-PA | CG9209 | 2 | behavioral response to ethanol |  |
|  | GB10339-PA | CG4533 | 10 | response to stress/heat |  |
|  | GB19264-PA | CG2647 | 1 | rhythmic behavior, response to temperature, response to light, courtship/mating behavior, circadian rhythm |  |
| Axonal | GB17380-PA | CG4609 | 9 | axonogenesis |  |
| function | GB16014-PA | CG4700 | 9 | axon guidance |  |
|  | GB14520-PA | CG3655 | 5 | axonal fasciculation |  |
|  | GB12585-PA | CG1511 | 1 | axonogenesis |  |
|  | GB15344-PA | CG10443 | 2 | motor axon guidance |  |
| Optic | GB14520-PA | CG3665 | 5 | Bolwig's organ morphogenesis |  |
| sensory | GB16014-PA | CG4700 | 9 | visual behavior |  |
|  | GB15257-PA | CG4917 | 9 | visual perception |  |
|  | GB11371-PA | CG3966 | 3 | rhodopsin biosynthesis, phototransduction |  |
|  | GB11326-PA | CG10546 | 3 | retinal binding |  |
|  | GB19218-PA | CG33553 | 8 | eye photoreceptor development |  |
|  | GB18494-PA | CG7399 | 6 | eye pigment biosynthesis |  |
| Vesicle | GB19359-PA | CG9012 | 9 | clathrin coat of synaptic vesicle |  |
| coating | GB19359-PA | CG9012 | 10 | clathrin vesicle coat |  |
| Kinase | GB11031-PA | CG32703 | 6 | MAPKKK cascade | 8 |
| pathway | GB11775-PA | CG9209 | 2 | MAPKKK cascade |  |
|  | GB13124-PA | CG4527 | 1 | MAPKKK cascade |  |
| Mushroom | GB11775-PA | CG9202 | 2 | mushroom body development |  |
| body | GB14520-PA | CG3665 | 5 | mushroom body development |  |
|  | GB14772-PA | CG7524 | 3 | mushroom body development |  |
| Neurogenesis | GB18507-PA | CG31641 | 5 | neurogenesis | 7 |
|  | GB15521-PA | CG32593 | 3 | neurogenesis |  |
|  | GB11432-PA | CG6378 | 7 | neurogenesis |  |
|  | GB12884-PA | CG17090 | 6 | neurogenesis |  |
|  | GB15344-PA | CG10443 | 2 | neurogenesis |  |
|  | GB17871-PA | CG6883 | 2 | neurogenesis |  |
|  | GB11115-PA | CG33141 | 2 | neurogenesis | 6 |
|  | GB19738-PA | CG10325 | 10 | neurogenesis |  |
|  | GB19218-PA | CG33553 | 8 | neurogenesis |  |
| Neurotrans- | GB11170-PA | CG6703 | 7 | neurotransmitter secretion |  |
| mission | GB11407-PA | CG5870 | 9 | neuromuscular synaptic transmission |  |
|  | GB11701-PA | CG7057 | 9 | neurotransmitter secretion |  |
|  | GB12095-PA | CG12095 | 7 | neurotransmitter transport |  |
|  | GB12564-PA | CG3985 | 5 | neurotransmitter secretion |  |
|  | GB13430-PA | CG33547 | 7 | neurotransmitter secretion |  |
|  | GB14520-PA | CG3665 | 5 | neuron recognition, neuromuscular junction development |  |
|  | GB15168-PA | CG5014 | 7 | neurotransmitter secretion, neuromuscular junction development |  |
|  | GB17817-PA | CG18069 | 5 | neuromuscular junction development |  |
|  | GB18122-PA | CG5226 | 9 | neurotransmitter transport |  |
|  | GB19372-PA | CG1732 | 5 | neurotransmitter transport |  |
|  | GB19979-PA | CG14994 | 7 | neuromuscular junction development, neurotransmitter biosynthesis, neurotransmitter receptor metabolism |  |
|  | GB11639-PA | CG7535 | 7 | GABA-A receptor activity |  |
|  | GB11487-PA | CG4322 | 3 | melatonin receptor activity |  |
|  | GB19359-PA | CG9012 | 10 | neurotransmitter secretion |  |
|  | GB16424-PA | CG3168 | 8 | neurotransmitter secretion |  |
|  | GB15327-PA | CG6917 | 2 | pheromone biosynthesis |  |
|  | GB14561-PA | CG33517 | 2 | dopamine D2 receptor-like receptor activity |  |
| Nervous | GB13770-PA | CG4535 | 7 | peripheral nervous system development |  |
| system | GB19218-PA | CG33553 | 8 | central nervous system development |  |
|  | GB19738-PA | CG10325 | 10 | peripheral nervous system development |  |
| Synaptic | GB14967-PA | CG18250 | 5 | dystroglycan complex, synapse formation |  |
| function | GB19979-PA | CG14994 | 7 | synaptogenesis |  |
|  | GB11170-PA | CG6703 | 7 | synaptic vesicle docking during exocytosis, synaptic vesicle targeting |  |
|  | GB11639-PA | CG7535 | 7 | postsynaptic membrane |  |
|  | GB11701-PA | CG7057 | 9 | synaptic vesicle coating |  |
|  | GB12095-PA | CG32490 | 7 | synaptic vesicle exocytosis |  |
|  | GB12564-PA | CG3985 | 5 | synaptic vesicle exocytosis |  |
|  | GB13430-PA | CG33547 | 7 | synaptic vesicle exocytosis |  |
|  | GB14520-PA | CG3665 | 5 | pre and postsynaptic membrane, regulation of synapse structure and function |  |
|  | GB15168-PA | CG5014 | 7 | synaptic vesicle transmission of nerve impulse, synaptic vesicle primint |  |
|  | GB11639-PA | CG7535 | 7 | nerve-nerve synaptic transmission |  |
|  | GB15431-PA | CG8884 | 9 | synapse | 7 |
|  | GB15431-PA | CG8884 | 7 | synaptic transmission |  |
|  | GB15745-PA | CG5618 | 7 | synaptic transmission |  |
|  | GB16014-PA | CG4700 | 9 | synaptic target inhibition | 7 |
|  | GB16424-PA | CG3168 | 8 | synaptic transmission of nerve impulse |  |
|  | GB16885-PA | CG1909 | 9 | synaptic transmission | 7 |
|  | GB17380-PA | CG4609 | 9 | transmission of nerve impulse | 7 |
|  | GB17817-PA | CG18069 | 5 | synaptic transmission |  |
|  | GB14561-PA | CG33517 | 2 | transmission of nerve impulse |  |
|  | GB19359-PA | CG9012 | 10 | synaptic vesicle coating |  |
|  | GB13617-PA | CG33555 | 2 | synaptic vesicle transport |  |
|  | GB15876-PA | CG5160 | 6 | synaptic transmission |  |
| Voltage channel | GB18284-PA | CG32684 | 3 | mannosyl-oligosaccharide 1  voltage-gated ion channel |  |
|  | GB17390-PA | CG9262 | 5 | voltage-gated potassium channel activity |  |
|  | GB18814-PA | CG12348 | 5 | voltage-gated potassium channel activity |  |
|  | GB12113-PA | CG6647 | 9 | voltage-gated ion channel activity |  |
|  | GB10325-PA | CG31116 | 1 | voltage-gated chloride channel activity |  |
|  | GB11639-PA | CG7535 | 5 | ligand-gated ion channels membrane |  |

Unless indicated otherwise, the same group assignment was found in the *ligustica* (*L*) data set. Most genes appear in more than one functional classification due to the multiple functional assignments. Red (blue) font identifies groups with descending (ascending) expression levels from day 0 (nurse) to day 17 (forager).
