# Supplementary material for: Semiparametric approach to characterize unique gene expression trajectories across time
Source: BMC Genomics. 2006 Sep 13;7:233. doi: 10.1186/1471-2164-7-233 (PMC1592090; doi:10.1186/1471-2164-7-233)
Supplement: Additional File 2 — List of 25 cDNAs with significant (P < 0.00001) linear or quadratic or cubic profiles from day 0 (nurse) to day 17 (forager), estimate of expression at each day in the ligustica (L) and mellifera (M) data sets, gene group, and the relative expression between forager and nurse (FvsN) bees reported by Whitfield et al. (2003). This table provides a list with the identification of 25 cDNAs with significant linear, quadratic or cubic trends across days, the estimated gene expression at each day, semiparametric group, and the relative expression between forager and nurse bees reported in Whitfield et al. (2003) for the L and M data sets. [file 1471-2164-7-233-S2.doc]

|  |  |  | Estimated normalized expression at day: | | | | |  |
| --- | --- | --- | --- | --- | --- | --- | --- | --- |
| cDNA | Data | Group | 0 | 4 | 8 | 12 | 17 | FvsN |
| BB160005A10H03 | *L* | 8 | 1.17 | 1.81 | 1.96 | 2.25 | 2.64 | 1 |
| BB160005A10H03 | *M* | 8 | 1.11 | 1.87 | 2.07 | 1.98 | 2.64 | 1 |
| BB160006A20F07 | *L* | 7 | 1.54 | 0.86 | 0.50 | 1.09 | 1.12 | 1 |
| BB160006A20F07 | *M* | 7 | 1.76 | 1.09 | 0.85 | 0.26 | 0.61 | 1 |
| BB160010A20H10 | *L* | 2 | -1.10 | -0.58 | 0.17 | 0.18 | 1.17 | 1 |
| BB160010A20H10 | *M* | 2 | -1.16 | -0.22 | -0.26 | -0.28 | 1.34 | 1 |
| BB160010B20F04 | *L* | 4 | 0.05 | 2.29 | 1.82 | 1.02 | 0.39 | -1 |
| BB160010B20F04 | *M* | 8 | -0.20 | 2.18 | 2.26 | 2.04 | 1.05 | -1 |
| BB160011B10C02 | *L* | 6 | 0.26 | 1.07 | 1.16 | 1.41 | 1.72 | 1 |
| BB160011B10C02 | *M* | 6 | 0.39 | 1.08 | 1.31 | 1.19 | 1.71 | 1 |
| BB160012B20F12 | *L* | 9 | 3.70 | 3.15 | 2.42 | 2.18 | 1.36 | -1 |
| BB160012B20F12 | *M* | 9 | 4.03 | 2.82 | 2.62 | 2.31 | 1.26 | -1 |
| BB160013A10H04 | *L* | 1 | -1.50 | -0.18 | -0.33 | -0.85 | -1.37 | -1 |
| BB160013A10H04 | *M* | 2 | -1.61 | -0.07 | 0.29 | 0.23 | -0.98 | -1 |
| BB160013A20G11 | *L* | 7 | 2.13 | 1.45 | 1.44 | 1.12 | 0.79 | -1 |
| BB160013A20G11 | *M* | 9 | 2.28 | 1.34 | 1.60 | 1.61 | 0.79 | -1 |
| BB160015A20B11 | *L* | 1 | -1.72 | -1.49 | -1.34 | -1.16 | -0.81 | 1 |
| BB160015A20B11 | *M* | 1 | -1.68 | -1.31 | -1.36 | -1.15 | -0.77 | 1 |
| BB160015A20E02 | *L* | 6 | -0.33 | -0.30 | 0.40 | 1.15 | 2.16 | 1 |
| BB160015A20E02 | *M* | 6 | -0.34 | -0.07 | -0.42 | 0.00 | 1.97 | 1 |
| BB160015B20E02 | *L* | 7 | 0.99 | 0.74 | 0.95 | 1.19 | 1.72 | 1 |
| BB160015B20E02 | *M* | 7 | 1.03 | 0.97 | 0.86 | 0.78 | 1.78 | 1 |
| BB160016A20C10 | *L* | 1 | -0.76 | -1.39 | -1.16 | -1.04 | -0.37 | 1 |
| BB160016A20C10 | *M* | 1 | -0.71 | -1.22 | -1.20 | -1.29 | -0.60 | 1 |
| BB160016B20F04 | *L* | 8 | 1.12 | 1.62 | 2.70 | 2.27 | 3.22 | 1 |
| BB160016B20F04 | *M* | 8 | 1.26 | 1.66 | 1.96 | 1.99 | 3.54 | 1 |
| BB160021B10B04 | *L* | 2 | -1.75 | 0.84 | -0.38 | -0.35 | -1.44 | -1 |
| BB160021B10B04 | *M* | 4 | -1.69 | 1.58 | 1.57 | 1.05 | -1.36 | -1 |
| BB160022A10D11 | *L* | 9 | 3.24 | 2.38 | 1.71 | 1.53 | 1.03 | -1 |
| BB160022A10D11 | *M* | 9 | 3.28 | 2.17 | 2.05 | 1.73 | 0.74 | -1 |
| BB160022B20A06 | *L* | 9 | 2.65 | 1.95 | 1.55 | 1.48 | 0.78 | -1 |
| BB160022B20A06 | *M* | 9 | 2.75 | 1.98 | 1.91 | 2.02 | 0.83 | -1 |
| BB160023B20C05 | *L* | 10 | 1.86 | 2.58 | 2.69 | 2.84 | 3.72 | 1 |
| BB160023B20C05 | *M* | 10 | 2.02 | 2.66 | 2.83 | 2.67 | 3.43 | 1 |
| BB170001A10D08 | *L* | 7 | 2.19 | 1.61 | 0.94 | 1.11 | 0.39 | -1 |
| BB170001A10D08 | *M* | 9 | 2.43 | 1.76 | 1.52 | 1.68 | 0.55 | -1 |
| BB170002B10H10 | *L* | 2 | -1.30 | -0.30 | -0.46 | -0.62 | -0.74 | -1 |
| BB170002B10H10 | *M* | 2 | -1.06 | -0.21 | -0.08 | -0.31 | -0.42 | -1 |
| BB170007B10D10 | *L* | 7 | 1.02 | 1.34 | 0.83 | 0.85 | 0.52 | -1 |
| BB170007B10D10 | *M* | 7 | 0.84 | 1.38 | 1.03 | 1.14 | 0.33 | -1 |
| BB170021B10F10 | *L* | 1 | -1.63 | -1.50 | -1.25 | -1.10 | -0.10 | 1 |
| BB170021B10F10 | *M* | 1 | -1.51 | -1.47 | -1.48 | -1.46 | -0.48 | 1 |
| BB170021B20G01 | *L* | 3 | -0.72 | -0.20 | -0.48 | -0.61 | -1.12 | -1 |
| BB170021B20G01 | *M* | 3 | -0.80 | -0.40 | -0.63 | -0.52 | -1.11 | -1 |
| BB170027B20E07 | *L* | 8 | 0.32 | 0.44 | 1.32 | 2.02 | 2.69 | 1 |
| BB170027B20E07 | *M* | 6 | 0.29 | 0.73 | 0.52 | 0.98 | 2.70 | 1 |
| BB170028A10C03 | *L* | 2 | -0.24 | -0.76 | -0.21 | -0.27 | 0.29 | 1 |
| BB170028A10C03 | *M* | 2 | -0.51 | -0.77 | -0.04 | -0.19 | 0.55 | 1 |
| BB170029B10B11 | *L* | 7 | 1.49 | 0.92 | 0.79 | 0.74 | 0.76 | -1 |
| BB170029B10B11 | *M* | 7 | 1.71 | 1.23 | 1.07 | 0.95 | 0.82 | -1 |

FvsN = 1 denotes cDNAs overexpressed in forager relative to nurse bees and -1 denotes cDNAs underexpressed in forager relative to nurse bees.
