# Supplementary material for: Semiparametric approach to characterize unique gene expression trajectories across time
Source: BMC Genomics. 2006 Sep 13;7:233. doi: 10.1186/1471-2164-7-233 (PMC1592090; doi:10.1186/1471-2164-7-233)
Supplement: Supplementary file 3 [file 1471-2164-7-233-S3.doc]

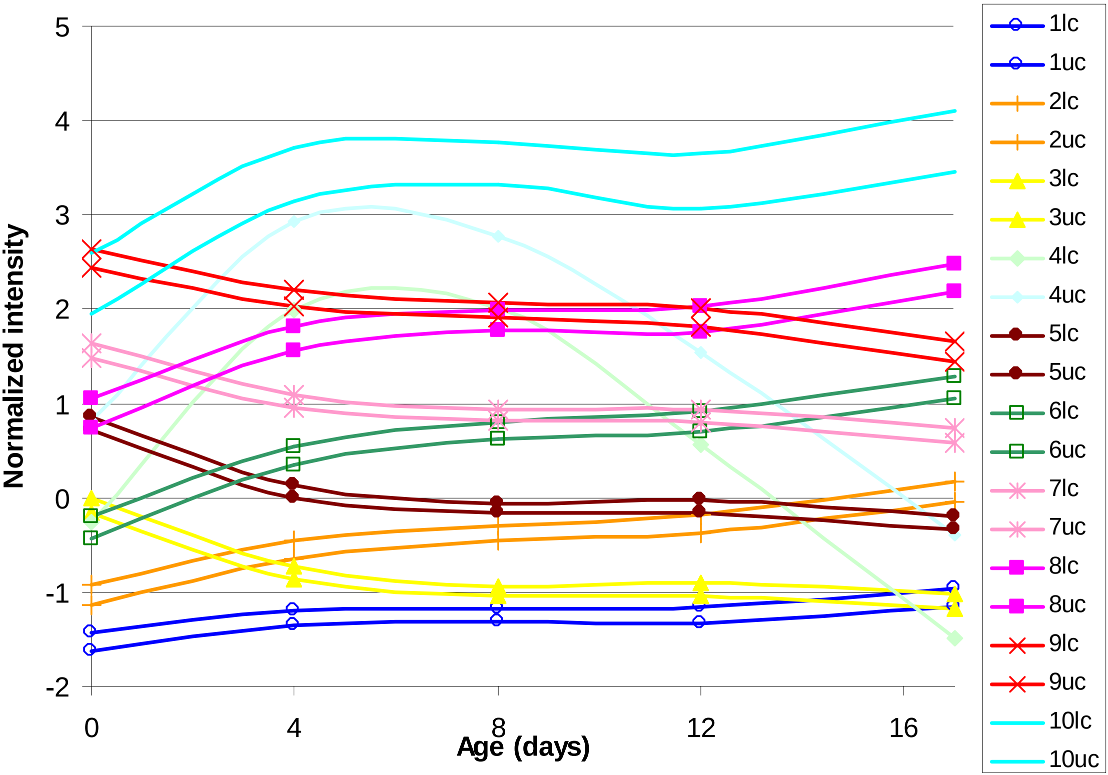

5
4
3
2
1
0
-1
-2
Normalized intensity
0
4
8
12
16
Age (days)
1lc
1uc
2lc
2uc
3lc
3uc
4lc
4uc
5lc
5uc
6lc
6uc
7lc
7uc
8lc
8uc
9lc
9uc
10lc
10uc
